# Exposure to mass media chronic health campaign messages and the uptake of non-communicable disease screening in Ghana

**DOI:** 10.1371/journal.pone.0302942

**Published:** 2024-05-31

**Authors:** Irenius Konkor, Elijah Bisung, Ophelia Soliku, Martin Ayanore, Vincent Kuuire

**Affiliations:** 1 Department of Geography, Geomatics and Environment, University of Toronto Mississauga, Mississauga, Canada; 2 School of Kinesiology and Health Studies, Queen’s University Kingston, Kingston, ON, Canada; 3 Department of Community Development, SD Dombo University of Business and Integrated Development Studies, Bamahu, Upper West Region, Ghana; 4 Department of Health Policy Planning and Management, School of Public Health, University of Health and Allied Sciences, Hohoe, Ghana; 5 Social and Behavioural Health Sciences Division, Dalla Lana School of Public Health, University of Toronto–St. George, Toronto, ON, Canada; SDD - University of Business and Integrated Development Studies, GHANA

## Abstract

The main goal of this study was to examine the relationship between exposure to mass media health campaign massages and the uptake of non-communicable diseases (NCDs) screening services in Ghana and whether this relationship differs by place of residence. Available evidence suggests a general low uptake of NCDs screening in developing country settings. Unfortunately, many NCDs evolve very slowly and are consequently difficult to detect early especially in situations where people do not screen regularly and in settings where awareness is low. In this study, we contribute to understanding the potential role of the media in scaling up NCDs screening in developing countries. We fitted multivariate logistic regression models to a sample of 1337 individual surveys which were collected at the neighborhood level in three Ghanaian cities. Overall, the results show that exposure to mass media chronic NCD health campaign messages was significantly associated with increased likelihood of screening for NCDs. The results further highlight neighborhood-level disparities in the uptake of NCDs screening services as residents of low-income and deprived neighborhoods were significantly less likely to report being screened for NCDs. Other factors including social capital, knowledge about the causes of NCDs and self-rated health predicted the likelihood of chronic NCDs screening. The results demonstrate mass media can be an important tool for scaling up NCDs screening services in Ghana and similar contexts where awareness might be low. However, place-based disparities need to be addressed.

## Introduction

According to the World Health Organization (WHO), chronic NCDs such as cancer, diabetes, cardiovascular diseases, and chronic respiratory diseases are responsible for about 41 million annual deaths–representing about 71% of all deaths globally [1]. Of this number, about 77% occur in low- and middle-income countries (LMICs) like Ghana [1]. The report further indicates that over 15 million NCD-related deaths are premature–occurring among individuals between the ages of 15 and 69 years and approximately 85% of these premature mortalities occur in LMICs. The high burden of NCDs in LMICs is consistent with the increasing prevalence of chronic NCD health outcomes. In Ghana, for example, research has shown a prevalence rate of 26.7% among a sample of Ghanaians with a mean age as low as 38 years [2]. A recent study using nationally representative data found hypertension prevalence to be as high as 29.7% [3]. Similarly, a systematic and meta-analyses found diabetes prevalence was about 6.4% [4, 5].

Even though most chronic NCDs are treatable if detected early, a fundamental set back is how most NCD patients especially in the context of developing country settings are unaware of their positive status until such diseases reach tipping points. Research, for instance, has shown that diabetes symptoms whether ophthalmic, neurological, or nephropathic mostly manifest only when they reach levels of permanent damage [6]. Yet, clinical studies in LMIC settings have found up to 50% and 73% of undiagnosed diabetes and hypertension cases respectively at the population level [7, 8]. Using a nationally representative sample, Tenkorang and colleagues found that over 44% of Ghanaians indicated they were not hypertensive when in deed they were based on systolic and diastolic blood pressure measures [9] indicating a mismatch between biometric data and undiagnosed self-reported data.

Indeed, many chronic NCDs develop slowly and imperceptible and are consequently difficult to detect early especially in situations where people do not undertake regular screening services. The high prevalence of undiagnosed chronic NCD cases in settings like Ghana could be attributed to low uptake of regular screening services underpinned by both individual level factors and structural determinants. At the structural level for example, poor doctor-to-population ratio of more than 6 times the WHO standard means significant sections of the population do not receive primary care where such conditions may be diagnosed [10]. Additionally, inadequate information availability on NCD risk factors and services could be crucial especially in an environment where chronic NCDs have not received the required attention at the population level. At a time when there is digitalization revolution in the Ghanaian context, mass media and internet platforms could be capitalized to sensitize the public and create awareness. This is particularly important because low awareness might be contributing to the high undiagnosed cases. The objective of this study, therefore, was to examine whether exposure to social and mass media campaign messages about chronic NCD health conditions was associated with the likelihood of NCDs screening in Ghana and whether the relationship differed geographically.

The Ghana Demographic and Health Survey (GDHS) has over the years recognized the importance of the media in broadcasting health messages and previous analysis of the data has shown access to broadcast media is generally high. For instance, 51% of men and 44% of women watch television at least once a week and 89% of men and 74% of women listen to the radio at least once a week [11]. More recent evidence has shown about 82% and 70% of Ghanaian adults are exposed to radio and television respectively at least once a week [12]. The print media is the one platform that witness the least exposure with only 16.7% of the population having exposure [12]. Given the overall high exposure to mass media, efforts have been made to promote healthy living through mass media platforms. One such campaign is the *Stop Aids Love Life HIV/AIDS* prevention campaign which has largely been assessed to be successful [13]. Given the taste for social media at the population level underpinned by widespread availability of smart phones and the growing availability of internet services albeit expensive, a well-coordinated health campaign could help improve chronic NCD awareness and best practices for improved health.

In fact, research studies elsewhere that examined the association between exposure to mass media smokeless tobacco warnings and intention/attempt to quit found that exposure to smokeless tobacco warnings in mass media promoted smokeless tobacco cessation [14]. The authors argue that health warnings through mass media could be beneficial to strategic policy programs aimed at reducing morbidity and mortality associated with smokeless tobacco use. Our study contributes to understanding how and in what ways social and mass media could be adopted as a tool for creating awareness and promoting chronic NCDs screening. Moreover, understanding geographical disparities in chronic NCDs screening could contribute to policy designs targeting specific locations for improved health outcomes. This is particularly crucial given the widening inequality between urban neighborhoods in Ghanaian cities. Recent political stability, economic growth and unequal economic opportunities in Ghana have conditioned widening socioeconomic gaps between the rich and the poor evident in emerging differential residential areas. Thus, the current economic landscape has contributed to emerging rich residential neighborhoods with high-quality services including healthcare, recreational centers and better schools [15]. On the other hand, increasing hardships among sections of the population with lower educational attainments and skills drive them to urban areas in search for greener pasture. Many of these new arrivals end up in already crowded and deprived neighborhoods that lack potable drinking water, toilet facilities, health facilities and other quality social amenities. We, therefore, expect that residents of deprived neighborhoods will be less inclined to screen for their NCDs status.

## Methods and data source

### Data and sampling strategy

This is a cross-sectional study, and a survey tool was used to collect the data from residents of nine neighborhoods between September and December 2021 across three cities (Wa; SSNIT = 102, Dokpong = 120, Wa-Zongo = 108, Tamale; Target = 127, Shiasego = 117, Gurugu = 118, and Accra; Dzorwulo = 193, Adabraka = 179, Gbegbeyise = 273) in Ghana. The cities were purposively selected to account for the different dimensions of the country’s development trajectories, an element we consider relevant for chronic NCD health outcomes. For example, although Accra has the smallest land area, it is the administrative capital and most populous and urbanized city in the country. Accra also has some of the best health facilities in the country. Tamale on the other hand has been considered the most rapidly urbanizing city in Ghana while Wa is one of the most deprived regional administrative capital cities in the country. Compared to the cities of Accra and Tamale, Wa has the poorest health facilities and skilled personnel ratio. In our sampling strategy, a multi-stage sampling was employed. After purposively selecting the cities, neighborhoods were categorized into high-, middle-, and low-income neighborhoods and one neighborhood was randomly selected from each category to administer the survey. In each of the selected neighborhoods, households were further systematically randomly selected (every fifth or tenth house depending on housing density) and an adult household member who was the last to celebrate his/her birthday was requested to respond to the survey. Residents were qualified to participate in the study if they were 18 years or older at the time of the fieldwork and resided in the selected neighborhood for at least a year prior the fieldwork. Overall, we collected data from 1385 individuals and the response rate was 92%. The analytic sample however was limited to 1337 individuals who were not missing on any key variable. The respondents were not offered any incentives to participate in the study.

The survey collected information on demographic and socio-economic characteristics of respondents, infectious diseases and chronic NCDs, exposure to chronic NCD health campaign messages from the mass media, chronic NCDs screening, neighborhood characteristics and knowledge about the causes of chronic NCD health conditions. The survey was pre-tested to ensure contextual validity and culturally appropriate questioning. Ethical clearance was granted by the Navrongo Health Research Center Institutional Review Board (#NHRCIRB424) in Ghana and written informed consent was given by all participants. The consent form was signed electronically during the survey and recorded in the database.

### Measurement of variables

The dependent variable in this analysis is “chronic NCDs screening” which was coded as either “0 = No” or “1 = Yes”. In the survey, respondents were asked if they had previously been screened for any chronic NCD health conditions and the responses included 1 = yes, 2 = no, 3 = don’t know and 4 = prefer not to answer. We, however, dropped responses to the latter two categories (don’t know = 26 and prefer not to answer = 5) due to lower cases. The main independent variable is exposure to mass media chronic NCD health campaign messages. We created this variable from a question that asked respondents “Have you heard or read about chronic NCD health information over the past 12 months from: any radio station, television station, print media, and social media” and the responses to each media platform were either yes or no. Hence, each media platform was created as a variable and coded as 0 = not exposed and 1 = exposed. Many media houses in Ghana also have social media handles such as Twitter (X) and/or Facebook. As a result, information from many television and radio stations are also relayed through these social media handles. Such a coordinated messaging makes it possible for anybody who has access to any of the above media outlets to have access to the same or similar media content. Consequently, we created a combined exposure variable by combing those who responded yes to any of the above media outlets as haven been exposed (1 = exposed) and those who indicated no to all as not being exposed (0 = not exposed). Because of our interest in understanding geographic variations in chronic NCDs screening, we measured neighborhood characteristics including neighborhood odor, neighborhood structural deprivation, and neighborhood economic deprivation. Guided by the literature on mass media exposure and health, we also measured relevant variables including neighborhood social capital, knowledge about the causes of NCDs, self-rated health, and socio-economic and demographic characteristics such as educational level, employment status, household wealth, age, gender, marital status, religion and city of residence.

Social capital, knowledge and the neighborhood variables were measured as scale variables using principal component analysis. We first tested the reliability of the items constituting each variable (see S1 Appendix) and found the alpha coefficients (neighborhood odor = 0.84, neighborhood structural deprivation = 0.79, and neighborhood economic deprivation = 0.89, social capital = 0.86 and NCDs knowledge = 0.72) to be high suggesting the items in each scale measured the intended construct and therefore appropriate for creating a scale. Household wealth was also created as an index by combing 23 household items (see S1 Appendix for details) that are a measure of wealth status in the study context using principal component analysis. The items produced an alpha value of 0.89 demonstrating they combined favorably and were consequently used to create an index, which was then categorized into quintiles.

### Analytic strategy

Three complementary analyses were conducted in this study. First, we conducted univariate analysis to observe the distribution of the dependent and independent variables. Secondly, we conducted logistic bivariate analysis to observe the relationship between the dependent variable and each of the independent variables. In the third category of analysis, we conducted multivariate logistic regression analysis for each of the four mass media outlets (radio, TV, print media, and social media). The dependent variable of the fifth model is the combined the media outlets. Results of the bivariate and multivariate analyses were exponentiated and presented as odds rations (ORs). Any variable or category with OR greater than one is interpreted as being more likely to report being screened for chronic NCDs and if the OR is less than one, that variable or category is reported as less likely. All analyses were conducted using Stata software the version 14.2.

## Results

### Univariate results

Results of the univariate analysis presented in Table 1 column 2 show that nearly 71% of respondents reported not having been previously screened for their NCDs status. In terms of exposure, however, 73.9% indicated being exposed to chronic NCD health campaigns on the radio, 74.6% through television stations, 24.5% through the print media and 42.6% through social media. Only 6.4% of the sample rated their health as fair/poor and a higher proportion (46%) rated their health as very good. About 14% of the sample did not attain formal education and 36.7% attained as high as tertiary education. About 31% of the sample reported being unemployed and the mean age of respondents was 41.7 years and a little more than half (51.5%) identified as women. Nearly two-thirds of the sample identified belonging to the Christian religious faith (63.1%) and 61.9% were married at the time of the survey.

**Table 1:** Distribution of the study variables and bivariate results of the relationship between the dependent and independent variables.

	Descriptive results	Bivariate results
Variable	Percent/Mean (Freq.)	ORs [95%CI]
NCDs screening		
No	70.5 (943)	‐‐‐‐‐‐‐‐‐‐‐
Yes	29.5 (394)	‐‐‐‐‐‐‐‐‐‐‐
**Mass media exposure**		
Not exposed	15.3 (204)	1.00[1.00,1.00]
Exposed	84.7 (1133)	2.63[1.76,3.93]^***^
**Radio station**		
Not exposed	26.1 (350)	1.00[1.00,1.00]
Exposed	73.9 (991)	1.36[1.03,1.80]^*^
**TV Station**		
Not exposed	25.4 (340)	1.00[1.00,1.00]
Exposed	74.6 (1001)	1.77[1.32,2.37]^***^
**Print media**		
Not exposed	75.5 (1013)	1.00[1.00,1.00]
Exposed	24.5 (328)	0.97[0.74,1.27]
**Social media**		
Not exposed	57.4 (769)	1.00[1.00,1.00]
Exposed	42.6 (572)	1.33[1.05,1.68]^*^
**NCDs knowledge**	0.0011203†	1.67[1.51,1.85]^***^
**Social capital**	-0.0003081†	1.08[1.02,1.15]^**^
**Self-rated health**		
Fair/poor	6.4 (86)	1.00[1.00,1.00]
Good	33.1 (443)	0.51[0.32,0.82]^**^
Very good	45.9 (614)	0.24[0.15,0.38]^***^
Excellent	14.5 (194)	0.22[0.12,0.37]^***^
**City of residence**		
Wa	24.7 (330)	1.00[1.00,1.00]
Tamale	27.1 (362)	2.53[1.82,3.53]^***^
Accra	48.2 (645)	1.21[0.88,1.65]
**Neighborhood type**		
High-income	31.6 (422)	1.00[1.00,1.00]
Middle-income	31.1 (416)	0.92[0.70,1.22]
Low-income	37.3 (499)	0.32[0.23,0.43]^***^
**Neighborhood odor**	0.0121189†	0.84[0.75,0.95]^**^
**Neighborhood structural deprivation**	0.0077758†	0.88[0.78,0.99]^*^
**Neighborhood economic deprivation**	-0.0022377†	1.01[0.90,1.14]
**Education level**		
No formal	14.0 (187)	1.00[1.00,1.00]
Primary	19.3 (258)	0.45[0.30,0.69]^***^
Secondary	30.0 (401)	0.47[0.32,0.69]^***^
Tertiary	36.7 (491)	0.98[0.69,1.38]
**Employment status**		
Employed	68.7 (918)	1.00[1.00,1.00]
Unemployed	31.3 (419)	0.90[0.69,1.16]
**Wealth quintile**		
Poorest	19.7 (263)	1.00[1.00,1.00]
Poorer	20.0 (267)	0.69[0.48,0.99]^*^
Middle	20.1 (269)	0.67[0.47,0.96]^*^
Rich	19.9 (266)	0.47[0.32,0.68]^***^
Richest	20.3 (272)	0.38[0.26,0.55]^***^
**Gender**		
Male	48.5 (649)	1.00[1.00,1.00]
Female	51.5 (688)	1.08[0.85,1.36]
**Age**		1.05[1.04,1.06]^***^
**Marital status**		
Never married	26.4 (353)	1.00[1.00,1.00]
Married	61.9 (828)	2.12[1.55,2.90]^***^
Divorced/widowed	11.7 (156)	4.91[3.23,7.46]^***^
**Religion**		
Other	1.4 (19)	1.00[1.00,1.00]
Christian	63.1 (844)	1.08[0.38,3.03]
Muslim	35.5 (474)	1.35[0.48,3.81]

*P<0.05

**P<0.01

***P<0.001

CI = confidence interval

† = summative mean

### Bivariate results

In the bivariate analysis (see Table 1), we found that respondents who were exposed to chronic NCD health campaign messages on radio [OR = 1.36, CI = 1.03,1.80], TV stations [OR = 1.77, CI = 1.32,2.37] and social media platforms [OR = 1.33, CI = 1.05,1.68] were more likely to screen for their NCD status compared to their counterparts who were not exposed. A unit increase in knowledge [OR = 1.67, CI = 1.51,1.85] about the causes of NCDs and neighborhood social capital [OR = 1.08, CI = 1.02,1.15] were observed to be positively associated with higher likelihood of screening for NCDs. Respondents who rated their health as very good [OR = 0.24, CI = 0.15,0.38] or excellent [OR = 0.22, CI = 0.12,0.37] were less likely to report getting screened for NCDs. Similarly, a unit increase in neighborhood structural deprivation [OR = 0.88, CI = 0.78,0.99] and neighborhood odor [OR = 0.84, CI = 0.75,0.95] were observed to be significantly associated with lower likelihood of NCDs screening. This observation is consistent with results of the neighborhood type as respondents from the low-income neighborhoods compared to those residing in high-income neighborhoods were less likely to screen for their NCDs status. In terms of city of residence, respondents from Tamale compared to those from Wa were more likely to screen for their NCDs status. We further observed that respondents who attained primary education [OR = 0.45, CI = 0.30,0.69] and secondary education [OR = 0.47, CI = 0.32,0.69] were significantly less likely to have screened for NCDs compared with those who did not attain formal education. In terms of wealth, however, respondents from the richest households were less likely to screen for their NCDs status but a unit increase in age [OR = 1.05, CI = 1.04,1.06] was associated with increased likelihood of screening for NCDs. Respondents who were widowed, divorced or separated [OR = 4.91, CI = 3.23,7.46], or married [OR = 2.12, CI = 1.55,2.90] were more likely compared with the never married respondents to report screening for their NCDs status.

### Multivariate results

#### Exposure to radio stations

The result of the multivariate analysis on exposure to radio stations and chronic NCDs screening was not statistically significant (see Table 2). However, a unit increase on knowledge about the causes of NCDs [OR = 1.59, CI = 1.41,1.79] and social capital [OR = 1.09, CI = 1.01,1.18] were found to be significantly associated with higher likelihood of screening for NCDs. Respondents who rated their health either as very good [OR = 0.25, CI = 0.14,0.44], or excellent [OR = 0.25, CI = 0.13,0.48] were observed to be less likely to screen for NCDs compared to those who rated their health as fair/poor. In terms of location, compared to respondents from the city of Wa, those from Tamale were more likely [OR = 2.33, CI = 1.48,3.67] to screen for their NCDs status. Respondents from neighborhoods classified as low-income neighborhoods [OR = 0.37, CI = 0.21,0.64] and those residing in neighborhoods deprived economically [OR = 0.76, CI = 0.61,0.94] were found to be less likely to screen for NCDs. In terms of socioeconomic characteristics, respondents who attained tertiary education [OR = 2.02, CI = 1.13,3.63] were more likely to screen for their NCDs status compared to their counterparts who did not receive formal education. A unit increase in age was also found to be significantly associated with increased likelihood of screening and women were more likely to screen for their NCDs status compared with men.

**Table 2:** Multivariate results of the relationship between the independent variables and NCDs screening.

	Model 1	Model 2	Model 3	Model 4	Model 5
Variable	Radio station	TV station	Print media	Social media	Combined media
	AOR [95% CI]	AOR [95% CI]	AOR [95% CI]	AOR [95% CI]	AOR [95% CI]
**Mass Media**					
Not exposed	1.00[1.00,1.00]	1.00[1.00,1.00]	1.00[1.00,1.00]	1.00[1.00,1.00]	1.00[1.00,1.00]
Exposed	1.21[0.84,1.75]	1.70[1.12,2.58]^*^	1.16[0.79,1.70]	1.51[1.03,2.20]^*^	2.77[1.61,4.76]^***^
**NCDs knowledge**	1.59[1.41,1.79]^***^	1.59[1.41,1.80]^***^	1.60[1.42,1.81]^***^	1.60[1.42,1.80]^***^	1.58[1.40,1.78]^***^
**Social capital**	1.09[1.01,1.18]^*^	1.09[1.01,1.18]^*^	1.10[1.01,1.18]^*^	1.09[1.01,1.18]^*^	1.09[1.00,1.18]^*^
**Self-rated health**					
Fair/poor	1.00[1.00,1.00]	1.00[1.00,1.00]	1.00[1.00,1.00]	1.00[1.00,1.00]	1.00[1.00,1.00]
Good	0.63[0.36,1.11]	0.62[0.35,1.09]	0.62[0.35,1.09]	0.60[0.34,1.05]	0.66[0.37,1.16]
Very good	0.25[0.14,0.44]^***^	0.25[0.14,0.44]^***^	0.24[0.14,0.43]^***^	0.24[0.13,0.42]^***^	0.26[0.15,0.47]^***^
Excellent	0.25[0.13,0.48]^***^	0.24[0.12,0.46]^***^	0.24[0.12,0.46]^***^	0.22[0.11,0.43]^***^	0.24[0.13,0.47]^***^
**City of residence**					
Wa	1.00[1.00,1.00]	1.00[1.00,1.00]	1.00[1.00,1.00]	1.00[1.00,1.00]	1.00[1.00,1.00]
Tamale	2.33[1.48,3.67]^***^	2.22[1.42,3.49]^***^	2.22[1.42,3.49]^***^	2.09[1.33,3.29]^**^	2.21[1.41,3.48]^***^
Accra	1.50[0.95,2.38]	1.45[0.92,2.31]	1.47[0.92,2.36]	1.46[0.92,2.33]	1.45[0.92,2.30]
**Neighborhood type**					
High-income	1.00[1.00,1.00]	1.00[1.00,1.00]	1.00[1.00,1.00]	1.00[1.00,1.00]	1.00[1.00,1.00]
Middle-income	1.08[0.68,1.71]	1.10[0.69,1.75]	1.08[0.68,1.70]	1.05[0.66,1.67]	1.17[0.73,1.86]
Low-income	0.37[0.21,0.64]^***^	0.40[0.23,0.70]^**^	0.36[0.20,0.62]^***^	0.36[0.21,0.63]^***^	0.42[0.24,0.75]^**^
**Neighborhood odor**	0.95[0.80,1.13]	0.94[0.79,1.11]	0.96[0.81,1.14]	0.96[0.81,1.14]	0.93[0.78,1.11]
**Neighborhood structure**	0.82[0.65,1.03]	0.82[0.65,1.04]	0.83[0.66,1.05]	0.80[0.63,1.02]	0.79[0.62,0.99]^*^
**Neighborhood economic**	0.76[0.61,0.94]^*^	0.76[0.62,0.95]^*^	0.75[0.60,0.93]^**^	0.76[0.61,0.93]^**^	0.78[0.63,0.96]^*^
**Education level**					
No formal	1.00[1.00,1.00]	1.00[1.00,1.00]	1.00[1.00,1.00]	1.00[1.00,1.00]	1.00[1.00,1.00]
Primary	1.21[0.70,2.11]	1.07[0.60,1.88]	1.26[0.73,2.19]	1.24[0.72,2.15]	1.03[0.59,1.82]
Secondary	1.21[0.71,2.06]	1.06[0.61,1.83]	1.25[0.74,2.12]	1.18[0.70,2.02]	1.01[0.58,1.74]
Tertiary	2.02[1.13,3.63]^*^	1.72[0.94,3.15]	2.05[1.14,3.67]^*^	1.82[1.00,3.30]^*^	1.68[0.92,3.05]
**Employment status**					
Employed	1.00[1.00,1.00]	1.00[1.00,1.00]	1.00[1.00,1.00]	1.00[1.00,1.00]	1.00[1.00,1.00]
Unemployed	0.82[0.59,1.14]	0.82[0.59,1.15]	0.81[0.58,1.13]	0.82[0.59,1.14]	0.83[0.60,1.16]
**Wealth quintile**					
Poorest	1.00[1.00,1.00]	1.00[1.00,1.00]	1.00[1.00,1.00]	1.00[1.00,1.00]	1.00[1.00,1.00]
Poorer	0.86[0.54,1.39]	0.91[0.57,1.46]	0.88[0.55,1.42]	0.94[0.58,1.52]	0.85[0.53,1.36]
Middle	0.77[0.44,1.34]	0.80[0.46,1.39]	0.78[0.45,1.37]	0.87[0.50,1.54]	0.76[0.44,1.31]
Richer	0.86[0.48,1.57]	0.93[0.51,1.69]	0.88[0.48,1.60]	1.00[0.54,1.85]	0.88[0.48,1.60]
Richest	0.54[0.27,1.08]	0.61[0.31,1.23]	0.54[0.27,1.06]	0.61[0.30,1.23]	0.62[0.31,1.22]
**Gender**					
Male	1.00[1.00,1.00]	1.00[1.00,1.00]	1.00[1.00,1.00]	1.00[1.00,1.00]	1.00[1.00,1.00]
Female	1.43[1.05,1.95]^*^	1.42[1.04,1.93]^*^	1.46[1.07,1.99]^*^	1.48[1.09,2.02]^*^	1.42[1.04,1.94]^*^
**Age**	1.05[1.04,1.06]^***^	1.05[1.04,1.06]^***^	1.05[1.04,1.06]^***^	1.05[1.04,1.07]^***^	1.05[1.04,1.06]^***^
**Marital status**					
Never married	1.00[1.00,1.00]	1.00[1.00,1.00]	1.00[1.00,1.00]	1.00[1.00,1.00]	1.00[1.00,1.00]
Married	0.76[0.49,1.17]	0.75[0.49,1.16]	0.77[0.50,1.19]	0.78[0.51,1.21]	0.76[0.49,1.17]
Divorced/widowed	0.96[0.51,1.81]	0.94[0.50,1.77]	0.97[0.51,1.83]	0.98[0.52,1.86]	0.93[0.49,1.76]
**Religion**					
Other	1.00[1.00,1.00]	1.00[1.00,1.00]	1.00[1.00,1.00]	1.00[1.00,1.00]	1.00[1.00,1.00]
Christian	2.31[0.71,7.50]	2.17[0.67,7.02]	2.38[0.73,7.75]	2.54[0.78,8.25]	2.00[0.61,6.60]
Muslim	1.99[0.60,6.57]	1.90[0.58,6.25]	2.09[0.63,6.91]	2.18[0.66,7.19]	1.80[0.54,6.03]
Number	1337.00	1337.00	1337.00	1337.00	1337.00
Intercept	0.03665	0.03139	0.03901	0.02931	0.01981
AIC	1281.85	1276.35	1282.30	1278.29	1268.09

*P<0.05

**P<0.01

***P<0.001

#### Exposure to TV stations

Unlike exposure to radio stations, respondents who were exposed to television stations were about 1.7 times likely [CI = 1.12,2.58] to screen for NCDs compared to their counterparts who were not exposed to chronic NCD health campaign messages on television. We further found that a unit increase on knowledge about the causes of NCDs [OR = 1.59, CI = 1.41,1.80] and social capital [OR = 1.09, CI = 1.01,1.18] were significantly associated with higher likelihood of screening for NCDs. Compared to respondents who rated their health as fair/poor, those who rated their health either as very good [OR = 0.25, CI = 0.14,0.44], or excellent [OR = 0.24, CI = 0.12,0.46] were less likely to screen for NCDs. Respondents from Tamale were about 2.2 times more likely [CI = 1.42,3.49] to screen for NCDs compared to their counterparts from Wa. Residing in low-income neighborhoods [OR = 0.40, CI = 0.23,0.70] and neighborhoods deprived economically [OR = 0.76, CI = 0.62,0.95] was associated with lower likelihood of screening for NCDs. Compared to men, women were more likely to screen for their NCDs status and a unit increase in age was associated with higher likelihood of screening for NCDs.

#### Exposure to the print media

Exposure to the print media did not show any significant difference in NCDs screening, but a unit increase in social capital [OR = 1.10, CI = 1.01,1.18] and NCDs knowledge [OR = 1.60, CI = 1.42,1.81] predicted higher likelihood of screening for NCDs. Consistent with the previous models, respondents who rated their health as very good [OR = 0.24, CI = 0.14,0.43], or excellent [OR = 0.24, CI = 0.12,0.46] were less likely to screen for NCDs. Similarly, respondents residing in neighborhoods classified as low-income neighborhoods [OR = 0.36, CI = 0.20,0.62] and neighborhoods deprived economically [OR = 0.75, CI = 0.60,0.93] were less likely to screen for NCDs. However, residents of Tamale [OR = 2.22, CI = 1.42,3.49] were significantly more likely to screen for NCDs compared to their counterparts from Wa. Women were also more likely to screen for NCDs compared to men and those who attained up to tertiary education were significantly more likely to screen for NCDs compared to those who did not receive formal education.

#### Exposure to social media

Results of model 4 show that exposure to social media was significantly associated with higher likelihood of screening for NCDs. For instance, respondents who were exposed to chronic NCD campaigns on social media platforms were about 1.5 times more likely [OR = 1.51, CI = 1.03,2.20] to screen for NCDs. Consistent with the previous models, a unit increase in social capital [OR = 1.09, CI = 1.01,1.18] and knowledge [OR = 1.60, CI = 1.42,1.80] about the causes of NCDs predicted increased likelihood of screening for NCDs. Participants who rated their health either as excellent [OR = 0.22, CI = 0.11,0.43] or very good [OR = 0.24, CI = 0.13,0.42] were less likely to screen for their NCDs status. The relationship between the geographic measures and NCDs screening remained consistent with results of the previous models. Similarly, women remained more likely to screen for their NCDs status compared to men and a unit increase in age predicted higher likelihood of screening for NCDs.

#### Combined media model

Model 5 (see Table 2) shows results of the combined media exposure and chronic NCDs screening in Ghana. The results show that respondents who were exposed to social and mass media campaign messages on chronic NCDs were more likely to screen for NCDs. In model 5, for example, those who were exposed were about 2.7 times more likely [CI = 1.61,4.76] to screen for their NCDs status compared to their counterparts who were not exposed. The relationships between knowledge about the causes of NCDs [OR = 1.58, CI = 1.40,1.78], social capital [OR = 1.09, CI = 1.00,1.18] and NCDs screening are consistent with results of the individual media outlets. Respondents who rated their health as very good [OR = 0.26, CI = 0.15,0.47] and excellent [OR = 0.24, CI = 0.13,0.47], however, were less likely to screen for NCDs. Compared to respondents residing in Wa, those residing in Tamale were more likely [OR = 2.21, CI = 1.41,3.48] to screen for their NCDs status. But residing in neighborhoods classified as low-income neighborhoods was associated with lower likelihood of screening compared to those residing in high-income neighborhoods. Similarly, we found that a unit increase in neighborhood structural deprivation [OR = 0.79, CI = 0.62,0.99] and neighborhood economic deprivation [OR = 0.78, CI = 0.63,0.96] were significantly associated with lower likelihood of screening for NCDs. A unit increase in age [OR = 1.05, CI = 1.04,1.06] was also observed to be associated with increased likelihood of screening and women [OR = 1.42, CI = 1.04,1.94] compared to men were more likely to screen for their NCDs status.

## Discussion

The high incidence of undiagnosed chronic NCD cases in many developing country settings including Ghana could partly be attributed to low uptake of screening services and more so for people living in deprived environments. In contributing to understanding the pattern of chronic NCDs screening, this study examined the relationship between exposure to social and mass media chronic NCD health campaign messages and the uptake of screening services in Ghana and whether this relationship differed by place of residence. Overall, the results show that exposure to mass media chronic NCD health campaign messages was significantly associated with increased likelihood of screening for NCDs. The results further highlight neighborhood-level disparities in the uptake of NCDs screening services as residents of low-income and deprived neighborhoods were significantly less likely to report being screened for NCDs. Other factors including social capital, knowledge about the causes of NCDs and self-rated health predicted the likelihood of chronic NCDs screening.

The observed positive relationship between exposure to mass media chronic NCD health campaign messages and the uptake of screening services is consistent with existing literature and offers empirical support to ongoing debates about the importance of mass media in disseminating relevant health information and addressing pressing public health issues [16–20]. Mass media has been widely used to reach higher proportions of populations to inform about emerging and ongoing issues including compelling health problems that might require the attention, cooperation, and behavioral responses of the public. Dating as far back as the 1700s, for example, mass media was used to promote inoculation for smallpox in the United States and later to promote healthy eating in the 1800s [21]. Empirical evidence, more recently, continue to show mass media has always been an important source of information about momentous health issues including HIV/AIDS, drug abuse, family planning, weight control and mammography [16–18, 21, 22]. Exposure to health promoting information and the ability to cogently digest such information often ignites a chain of cognitive processes and reactions motivated by self-desire to remain healthy for as long as possible. Such exposure also improves awareness and conscientiousness about the benefits of observing certain healthy practices.

Previous researchers have consequently noted the potential of mass media as a health campaign tool lies in their ability to widely disseminate well crafted messages to audiences repeatedly, over time, in an incidental manner and at a lower cost [23]. While the health impacts of mass media campaigns may be inconsistently realized across locations and even between health issues, appropriate framing of chronic NCD health massages in the Ghanaian context and similar developing country settings could prove effective. The framing of the problem as well as the delivery style are particularly important especially given the proportion of reported exposure to NCDs campaign messages (84.8%) versus those who have screened (29.4%) for their NCDs status in this study. The gap in exposure and screening could consequently be due to contextual factors. For example, previous research in Ghana has shown that the time allocated for health programs on mass media platforms are usually short, which makes it difficult for listeners to learn enough from such programs [24]. Other problems are that the health programs are either presented late in the day or the content is presented using medical jargon that is not readily comprehensible by the ordinary listener [24]. Yet, previous studies have shown that the nature of information conveyed by media outlets, the coverage and the presentation techniques can have powerful effects on knowledge, behavioral changes, and attitudes [25–27] and health campaign programs should be designed targeting these key elements.

The results further highlight geographic disparity in the uptake of chronic NCDs screening services in Ghana. Participants of the more deprived and low-income residential neighborhoods, for example, were less likely to screen for their chronic NCDs status compared to their counterparts from the less deprived and high-income neighborhoods. Annim and Colleages [28] in an earlier study noted that within district inequity in Ghana contributes more to national inequality than between district inequity. Inferentially, inequity within towns and cities in Ghana is more endemic than inequities between these geographic locations. It is consequently not surprising to observe disparities in the uptake of NCDs screening services between neighborhoods. Even though the country has achieved some macroeconomic gains in the past few decades, the gains have not been translated into improving economic wealth at the individual level [29, 30] and in exceptional cases the few people particularly those connected politically benefit from these economic gains. The masses are therefore left with limited opportunities and a significant proportion of these people live in the more deplorable parts of the cities.

As Eriksson [31] previously noted “information constitutes an essential basis for actions *and that a crucial component* of social capital is the potential information embedded in social relations” (pp.2). Besides information sharing, social/peer influence is another relevant pathway between social network and health [32]. While health knowledge and information sharing among neighbors in the study neighborhoods could be an important determinant of NCDs screening, the positive influence of networking with people who engage in preventative practices including NCD screening could have a trickle-down effect. Putnam [33] has argued that even though social capital could be a private good, it is at the same time a collective and nonexclusive good at the community level. This is because living in a high social capital area could be beneficial even for people who do not have strong social connections or knowledge about certain health conditions since spill-over benefits could accrue to such individuals.

### Limitations

Even though these findings offer important policy pointers for scaling up chronic NCDs screening in Ghana, there are some noteworthy limitations. First, because the study design is cross-sectional, the relationship between mass media exposure and chronic NCDs screening can best be described as associations. Secondly, there is the possibility of reporting bias as the data in the study were obtained from self-report. Future researchers interested in understanding the role of the media and preventative health practices could implement experimental study design that involves exposure and control groups to examine any “real” differences in their uptake of chronic NCDs screening services. Finally, a onetime exposure might not be enough to trigger any positive response, but our exposure variable did not differentiate the number/duration of exposure and that could partly explain why a lower proportion of the sample screened even though a higher proportion reported being exposed. That being said, the packaging and style of presentation could be influential on the overall acceptability of the information. Wakefield and Colleagues [23] have noted that media campaigns can fall short due to numerous factors including inadequate funding, fractured and cluttered media environment, age-inappropriate content, and the use of homogeneous strategies to reach heterogeneous audience and these factors must be considered during the design stage to achieve maximum results.

## Conclusion

This study examined the connections between exposure to social and mass media chronic NCDs campaign messages and the uptake of NCDs screening in Ghana. The results showed that responds who were exposed were significantly more likely to screen for their NCDs status compared to those who were not exposed. However, respondents from deprived neighborhoods were less likely to screen for NCDs. Despite the potential limitations discussed above, our study is important and offers some policy pathways for improving NCDs screening in Ghana and similar LMIC settings. The findings, for example, suggest that a well composed health media campaign program could improve NCDs screening, which will then lead to early detection and improved treatment outcomes at a reduced cost for individuals and families. Such campaign messages, however, should be presented in local languages to be easily digested by local audiences. The study further demonstrates the need to consciously address neighborhood disparities in the Ghanaian context. High unemployment rate coupled with high cost of living in many Ghanaian cities have led to the emergence of deplorable settlements and the expansion of existing slums which produce conditions for poor health outcomes [34]. Importantly, residents of such spaces do not often have the financial resources to access health service and mostly do not even consider voluntary screening a priority until they are too sick to go about their daily activities. Ongoing media campaigns could be complemented with free screening and counselling services to offer marginalized groups the opportunity to screen. In fact, if media campaigns focus on approaches for which the target population lack the resources to address, the intended objective might never be realized. To this end, the current pro-poor national health insurance program could be retooled and resourced to cover screening costs and medication services for NCDs. In some jurisdictions community health workers have been resourced and deployed to screen people at their homes [35, 36] and this strategy could be adapted and implemented in Ghana to reach out to underserved communities and help overcome bureaucratic procedures at tertiary health facilities where chronic NCD health services are commonly provided.

Finally, given the positive relationship between social capital and NCDs screening, existing social networks could be empowered to be agents of NCD campaign messages. The advantage is that strong social networks already exist at all levels within the Ghanaian society. One of such strong social organization is the religious institution. Many Ghanaians consider themselves as religious people and have trust in their religious leaders and empowering them as agents with a mandate to include chronic NCD health teachings to their sermons could prove a worthwhile bargain. Other local societal unions could as well be empowered to promote healthy practices.

## Supporting information

S1 Appendix Measures of scale variables. (DOCX)
