# Correction: Exposure to mass media chronic health campaign messages and the uptake of non-communicable disease screening in Ghana

**DOI:** 10.1371/journal.pone.0326688

**Published:** 2025-06-23

**Authors:** Irenius Konkor, Elijah Bisung, Ophelia Soliku, Martin Ayanore, Vincent Kuuire

The Data Availability statement and the ethics statement in the Methods and data source section of this article [1] are incorrect.

The correct Data Availability statement is:

Location: University of Toronto Dataverse DOI: https://doi.org/10.5683/SP3/GHHINH.

The correct ethics statement is:

Ethical clearance was granted by the Navrongo Health Research Center Institutional Review Board (#NHRCIRB424) in Ghana and the University of Toronto Social Sciences, Humanities and Education Research Ethics Board (RIS Protocol Number 41065) and written informed consent was given by all participants. The consent form was signed electronically during the survey and recorded in the database.
