# Supplementary material for: Exposure to mass media chronic health campaign messages and the uptake of non-communicable disease screening in Ghana
Source: PLoS One. 2024 May 31;19(5):e0302942. doi: 10.1371/journal.pone.0302942 (PMC11142513; doi:10.1371/journal.pone.0302942)
Supplement: S1 Appendix — (DOCX) [file pone.0302942.s001.docx]

**Appendix A: Measures of scale variables**

| **Table 1. Neighborhood economic deprivation. Response options: Strongly disagree, Disagree, Neutral, Agree, Or Strongly agree** | |
| --- | --- |
| **Item No.** | **Item** |
| 1 | Most of the people in my neighborhood are college and university graduates |
| 2 | The people in my neighborhood have very good jobs |
| 3 | Most people in my neighborhood earn decent income |
| 4 | Most adults in my neighborhood have cars/motorbikes |
| 5 | Most individuals in my neighborhood have their own rooms |
| 6 | Most households in my neighborhood can buy their needs without external help |

**Table 1a. Correlation matrix: neighborhood economic deprivation index**

| Item | 1 | 2 | 3 | 4 | 5 | 6 |  |
| --- | --- | --- | --- | --- | --- | --- | --- |
| 1 | 1.000 |  |  |  |  |  |  |
| 2 | 0.808 | 1.000 |  |  |  |  |  |
| 3 | 0.766 | 0.894 | 1.000 |  |  |  |  |
| 4 | 0.504 | 0.550 | 0.539 | 1.000 |  |  |  |
| 5 | 0.417 | 0.477 | 0.463 | 0.657 | 1.000 |  |  |
| 6 | 0.397 | 0.430 | 0.430 | 0.540 | 0.650 | 1.000 |  |
|  | | | | | | | |

**Table 1b. Principal components with Eigenvalues and Proportion of Variance:** **neighborhood economic deprivation index**

| Component | Eigenvalue | Difference | Proportion | Cumulative |
| --- | --- | --- | --- | --- |
| Comp1 | 3.860 | 2.821 | 0.643 | 0.643 |
| Comp2 | 1.040 | 0.595 | 0.173 | 0.817 |
| Comp3 | 0.445 | 0.141 | 0.074 | 0.891 |
| Comp4 | 0.304 | 0.054 | 0.051 | 0.941 |
| Comp5 | 0.250 | 0.149 | 0.042 | 0.983 |
| Comp6 | 0.101 | . | 0.017 | 1.000 |

**Table 1c. Principal component loadings (eigenvectors): neighborhood economic deprivation index**

| Item | Comp1 | Comp2 | Comp3 | Comp4 | Comp5 | Comp6 | Unexplained |
| --- | --- | --- | --- | --- | --- | --- | --- |
| 1 | 0.419 | -0.383 | 0.085 | 0.099 | 0.800 | 0.143 | 0 |
| 2 | 0.449 | -0.366 | 0.048 | -0.103 | -0.267 | -0.761 | 0 |
| 3 | 0.442 | -0.360 | 0.065 | -0.101 | -0.512 | 0.631 | 0 |
| 4 | 0.398 | 0.295 | -0.708 | 0.501 | -0.054 | -0.001 | 0 |
| 5 | 0.379 | 0.500 | -0.124 | -0.755 | 0.142 | 0.028 | 0 |
| 6 | 0.353 | 0.503 | 0.685 | 0.385 | -0.062 | -0.020 | 0 |

| **Table 2. Neighborhood social capital. Response options: Strongly disagree, Disagree, Neutral, Agree, Or Strongly agree** | |
| --- | --- |
| **Item No.** | **Item** |
| 1 | This is a close-knit or unified neighborhood |
| 2 | People in my neighborhood are willing to help one another |
| 3 | People in my neighborhood generally get along with each other |
| 4 | People in my neighborhood can be trusted |
| 5 | People in my neighborhood share common values |
| 6 | People in my neighborhood share valuable health information |
| 7 | People in my neighborhood sometimes share food with each other |
| 8 | My neighbors take care of my home when I am away |

**Table 2a. Correlation matrix: neighborhood social capital index**

| Item | 1 | 2 | 3 | | 4 | | 5 | | 6 | | 7 | | 8 |
| --- | --- | --- | --- | --- | --- | --- | --- | --- | --- | --- | --- | --- | --- |
| 1 | 1.0000 |  | |  | |  |  |  |  |  |  |  |  |
| 2 | 0.5357 | 1.0000 |  | | |  | |  |  |  |  |  |  |
| 3 | 0.5105 | 0.7310 | 1.0000 | |  | | |  | |  |  |  |  |
| 4 | 0.1600 | 0.3888 | 0.3807 | | 1.0000 | |  | | |  | |  |  |
| 5 | 0.4037 | 0.4364 | 0.4497 | | 0.4655 | | 1.0000 | |  | |  | |  |
| 6 | 0.3153 | 0.4833 | 0.5164 | | 0.5228 | | 0.6126 | | 1.0000 | |  | |  |
| 7 | 0.2600 | 0.4641 | 0.4704 | | 0.4532 | | 0.5009 | | 0.5781 | | 1.0000 | |  |
| 8 | 0.3423 | 0.4007 | 0.3515 | | 0.3442 | | 0.5018 | | 0.4124 | | 0.5357 | | 1.0000 |
|  | | | | | | | | | | |  | |  |

**Table 2b. Principal components with Eigenvalues and Proportion of Variance:** **neighborhood economic deprivation index**

| Component | Eigenvalue | Difference | Proportion | Cumulative |
| --- | --- | --- | --- | --- |
| Comp1 | 4.16424 | 3.11083 | 0.5205 | 0.5205 |
| Comp2 | 1.05341 | 0.318028 | 0.1317 | 0.6522 |
| Comp3 | 0.735383 | 0.155704 | 0.0919 | 0.7441 |
| Comp4 | 0.579679 | 0.0815955 | 0.0725 | 0.8166 |
| Comp5 | 0.498084 | 0.118729 | 0.0623 | 0.8788 |
| Comp6 | 0.379355 | 0.0510678 | 0.0474 | 0.9263 |
| Comp7 | 0.328287 | 0.0667255 | 0.0410 | 0.9673 |
| Comp8 | 0.261562 | . | 0.0327 | 1.0000 |

**Table 2c. Principal component loadings (eigenvectors): neighborhood social capital index**

| Item | Comp1 | Comp2 | Comp3 | Comp4 | Comp5 | Comp6 | Comp7 | Comp8 | Unexplained |
| --- | --- | --- | --- | --- | --- | --- | --- | --- | --- |
| 1 | 0.2928 | 0.5990 | 0.2529 | 0.3839 | 0.1482 | 0.5603 | 0.0781 | 0.0470 | 0 |
| 2 | 0.3808 | 0.3727 | -0.2301 | -0.2870 | -0.1114 | -0.2636 | -0.0267 | -0.7058 | 0 |
| 3 | 0.3793 | 0.3529 | -0.3161 | -0.2431 | -0.0945 | 0.2867 | -0.0875 | 0.6883 | 0 |
| 4 | 0.3135 | -0.4203 | -0.4502 | 0.1465 | -0.6729 | -0.2084 | -0.0254 | 0.0637 | 0 |
| 5 | 0.3738 | -0.1777 | 0.1886 | 0.5847 | 0.1878 | 0.3621 | -0.5297 | -0.0658 | 0 |
| 6 | 0.3829 | -0.2422 | -0.1920 | 0.5847 | -0.4842 | -0.0317 | 0.6852 | -0.0630 | 0 |
| 7 | 0.3657 | -0.2870 | 0.1323 | -0.4842 | 0.3307 | 0.5305 | -0.3739 | -0.0366 | 0 |
| 8 | 0.3272 | -0.1690 | 0.7004 | -0.2537 | 0.3532 | 0.2783 | 0.3082 | 0.1102 | 0 |

| **Table 3. Neighborhood odor. Response options: Strongly disagree, Disagree, Neutral, Agree, Or Strongly agree** | |
| --- | --- |
| **Item No.** | **Item** |
| 1 | There are lots of trash and litter in my neighborhood |
| 2 | There is a lot of noise in my neighborhood |
| 3 | There is open defecation in my neighborhood |
| 4 | There is stench from the gutters in my neighborhood |

**Table 3a. Correlation matrix: neighborhood odor index**

| Item | 1 | 2 | 3 | | 4 | |
| --- | --- | --- | --- | --- | --- | --- |
| 1 | 1.0000 |  | |  | |  |
| 2 | 0.4762 | 1.0000 |  | | |  |
| 3 | 0.5589 | 0.4960 | 1.0000 | |  |  |
| 4 | 0.5686 | 0.5600 | 0.7002 | | 1.0000 | |

**Table 3b. Principal components with Eigenvalues and Proportion of Variance:** **neighborhood odor index**

| Component | Eigenvalue | Difference | Proportion | Cumulative |
| --- | --- | --- | --- | --- |
| Comp1 | 2.68619 | 2.14436 | 0.6715 | 0.6715 |
| Comp2 | 0.541833 | 0.0631085 | 0.1355 | 0.8070 |
| Comp3 | .478724 | 0.185473 | 0.1197 | 0.9267 |
| Comp4 | .293251 | . | 0.0733 | 1.0000 |

**Table 3c. Principal component loadings (eigenvectors): neighborhood social capital index**

| Item | Comp1 | Comp2 | Comp3 | Comp4 | Unexplained |
| --- | --- | --- | --- | --- | --- |
| 1 | 0.4823 | -0.3504 | 0.8029 | -0.0034 | 0 |
| 2 | 0.4652 | 0.8677 | 0.0999 | 0.1442 | 0 |
| 3 | 0.5176 | -0.3325 | -0.4534 | 0.6449 | 0 |
| 4 | 0.5320 | -0.1174 | -0.3740 | -0.7505 | 0 |

| **Table 4. NCDs knowledge. Response options: Disagree, or Agree** | |
| --- | --- |
| **Item No.** | **Item** |
| 1 | Poor diet is a major risk factor for chronic disease |
| 2 | Lack of exercise increases the risk of chronic disease |
| 3 | Too much alcohol consumption can cause chronic disease |
| 4 | Too much sodium/salt consumption can cause chronic disease |
| 5 | Smoking is a major risk factor for chronic disease |
| 6 | People who consistently add vegetables in their diets can develop chronic disease |
| 7 | Low fruit consumption can reduce my risk of chronic disease |

**Table 4a. Correlation matrix: NCDs knowledge index**

| Item | 1 | 2 | 3 | | 4 | | 5 | | 6 | | 7 | |
| --- | --- | --- | --- | --- | --- | --- | --- | --- | --- | --- | --- | --- |
| 1 | 1.0000 |  | |  | |  |  |  |  |  |  |  |
| 2 | 0.5539 | 1.0000 |  | | |  | |  |  |  |  |  |
| 3 | 0.1294 | 0.2014 | 1.0000 | |  | | |  | |  |  |  |
| 4 | 0.0125 | 0.0171 | -0.0889 | | 1.0000 | |  | | |  | |  |
| 5 | 0.0631 | 0.0534 | 0.0780 | | -0.0674 | | 1.0000 | |  | |  | |
| 6 | -0.0209 | -0.0334 | 0.0054 | | 0.4040 | | -0.1312 | | 1.0000 | |  | |
| 7 | -0.0671 | -0.0865 | -0.0111 | | 0.4350 | | -0.1514 | | 0.7579 | | 1.0000 | |
|  | | | | | | | | | | |  | |

**Table 4b. Principal components with Eigenvalues and Proportion of Variance:** **NCDs knowledge index**

| Component | Eigenvalue | Difference | Proportion | Cumulative |
| --- | --- | --- | --- | --- |
| Comp1 | 2.14639 | .516717 | 0.3066 | 0.3066 |
| Comp2 | 1.62967 | .648053 | 0.2328 | 0.5394 |
| Comp3 | .981619 | .0558633 | 0.1402 | 0.6797 |
| Comp4 | .925755 | .286729 | 0.1323 | 0.8119 |
| Comp5 | .639026 | .200242 | 0.0913 | 0.9032 |
| Comp6 | .438784 | .20003 | 0.0627 | 0.9659 |
| Comp7 | .238754 | . | 0.0341 | 1.0000 |

**Table 4c. Principal component loadings (eigenvectors): NCDs knowledge index**

| Item | Comp1 | Comp2 | Comp3 | Comp4 | Comp5 | Comp6 | Comp7 | Unexplained |
| --- | --- | --- | --- | --- | --- | --- | --- | --- |
| 1 | -0.1324 | 0.6348 | -0.2483 | 0.1512 | -0.1852 | 0.6786 | 0.0136 | 0 |
| 2 | -0.1452 | 0.6512 | -0.1902 | 0.0452 | 0.0016 | -0.7175 | 0.0433 | 0 |
| 3 | -0.0918 | 0.3274 | 0.5890 | -0.6630 | 0.2897 | 0.1182 | -0.0056 | 0 |
| 4 | 0.4516 | 0.1500 | -0.0454 | 0.3375 | 0.8059 | 0.0769 | -0.047 | 0 |
| 5 | -0.1940 | 0.0848 | 0.7210 | 0.6453 | -0.1324 | -0.0335 | 0.0172 | 0 |
| 6 | 0.5876 | 0.1562 | 0.1320 | -0.0509 | -0.3648 | -0.0576 | -0.6884 | 0 |
| 7 | 0.6051 | 0.1084 | 0.1263 | -0.0523 | -0.2858 | -0.0179 | 0.7221 | 0 |

**Table 5. Household wealth index items**

| Automobile | Motorbike | Motor-tricycle | Deep freezer | Fridge | Tap water |
| --- | --- | --- | --- | --- | --- |
| Electricity | Electric iron | Ceiling fan | Standing fan | Shower | Tiled floor |
| Satellite television | Internet at home | Smart phone | Tablet | Laptop | Block house |
| Lawn | Water closet toilet | Kitchen room | Gas cooker | Fence wall |  |
